# A comparative analysis of the foamy and ortho virus capsid structures reveals an ancient domain duplication

**DOI:** 10.1186/s12900-017-0073-0

**Published:** 2017-04-04

**Authors:** William R. Taylor, Jonathan P. Stoye, Ian A. Taylor

**Affiliations:** 1 Computational Cell and Molecular Biology Laboratory, Francis Crick Institute, Midland Road, London, NW1 1AT UK; 2 Retrovirus-Host Interactions Laboratory, Francis Crick Institute, Midland Road, London, NW1 1AT UK; 3 Macromolecular Structure Laboratory, Francis Crick Institute, Midland Road, London, NW1 1AT UK

**Keywords:** Virus capsid structure, Foamy virus evolution, Protein structure comparison

## Abstract

**Background:**

The *Spumaretrovirinae* (foamy viruses) and the *Orthoretrovirinae* (e.g. HIV) share many similarities both in genome structure and the sequences of the core viral encoded proteins, such as the aspartyl protease and reverse transcriptase. Similarity in the *gag* region of the genome is less obvious at the sequence level but has been illuminated by the recent solution of the foamy virus capsid (CA) structure. This revealed a clear structural similarity to the orthoretrovirus capsids but with marked differences that left uncertainty in the relationship between the two domains that comprise the structure.

**Methods:**

We have applied protein structure comparison methods in order to try and resolve this ambiguous relationship. These included both the DALI method and the SAP method, with rigorous statistical tests applied to the results of both methods. For this, we employed collections of artificial fold ’decoys’ (generated from the pair of native structures being compared) to provide a customised background distribution for each comparison, thus allowing significance levels to be estimated.

**Results:**

We have shown that the relationship of the two domains conforms to a simple linear correspondence rather than a domain transposition. These similarities suggest that the origin of both viral capsids was a common ancestor with a double domain structure. In addition, we show that there is also a significant structural similarity between the amino and carboxy domains in both the foamy and ortho viruses.

**Conclusions:**

These results indicate that, as well as the duplication of the double domain capsid, there may have been an even more ancient gene-duplication that preceded the double domain structure. In addition, our structure comparison methodology demonstrates a general approach to problems where the components have a high intrinsic level of similarity.

**Electronic supplementary material:**

The online version of this article (doi:10.1186/s12900-017-0073-0) contains supplementary material, which is available to authorized users.

## Background

Taxonomically, the *Orthoretrovirinae* (orthoretroviruses) and *Spumaretrovirinae* ^1^ (spumaviruses) make up the two subfamilies of *Retroviridae*. They share many similarities, including overall genome structures with gag, pol and env genes encoding proteins for replication and life cycles involving reverse transcription and integration into the chromosomes of infected cells. However, there are also a number of differences distinguishing these viral subfamilies, including finer details of genome organisation, the absence of a Gag-Pol fusion protein in spumaviruses and the timing of reverse transcription [1].

Gag is the major structural protein of both Ortho and Foamy viruses and is responsible for many of the differences and similarities between the viral subfamilies. Ortho and Foamy viral Gags are required for particle assembly, budding from the cell, reverse transcription and delivery of the viral nucleic acid into the newly infected cell. However, there are a number of striking differences including how the Gag precursor is targeted to the cell membrane, the absence of a Major Homology Region and Cys-His box in Foamy viruses and very different patterns of processing during viral maturation [2]. In all Ortho viruses, Gag is proteolytically cleaved to form distinct, well-studied proteins, matrix (MA), capsid (CA) and nucleocapsid (NC), found in mature virions, whilst in spumaviruses Gag processing to remove a C-terminal peptide occurs only in a fraction of the Gag molecules [3].

Structural information regarding foamy virus Gag has been limited to the crystal structure of the N-terminal Env binding region of Prototypic Foamy virus (PFV) Gag (PFV-Gag-NtD) that although maintaining some of the function of orthoretrivial MA shared no structural similarity [4]. However, more recently the solution NMR structure of the PFV Gag central CA domains has shed new light on the relationship between ortho and spumaviruses. It reveals that the CA structures of both viral subfamilies share a common protein fold, implying that their Gag proteins may be evolutionarily related [5].

However, an intriguing aspect of this relationship was an ambiguity in the degree of relatedness between the CA domains of the Gag proteins, with the Spumaretroviral CA domains, NtDCEN and CtDCEN, appearing almost equally similar to either the amino- (CA-NtD) or carboxy-terminal (CA-CtD) domains of the orthoretroviruses. With small domains that share a high degree of background similarity, particularly those composed entirely of *α*-helices, it is very difficult to evaluate the significance of their structural relationships as chance combinations of a few helices can give rise to an apparently convincing overlaps with a low RMSD.

In this paper, we now investigate and clarify the nature of the relationship between these capsid domains and discuss its evolutionary implications. Our work provides a demonstration of a general approach to the resolution of difficult comparison problems in which the proteins share a high intrinsic level of similarity.

## Results

### Full-length comparison

**Fig. 1:**
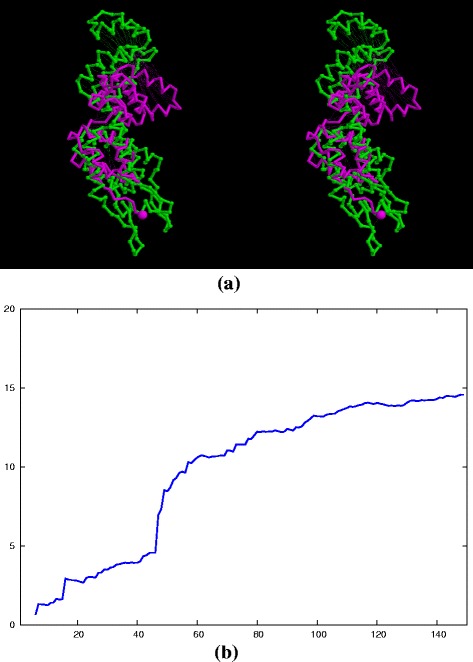
Full ortho/foamy virus capsid superposition. The superposed structures are shown in part (**a**) as a stereo pair, coloured as *green* = ortho virus (HIV, PDB code: 3nte-A) and *magenta* = foamy virus capsid. (The amino terminus is marked by a small sphere). Part (**b**) shows the cumulative RMSD plot for this superposition which plots the RMSD value (*Y*-axis) for increasingly larger sets of residues as ranked by their SAP similarity score (*X*-axis). The sharp rise in this trace marks the transition into subsets that include positions from the displaced domain

To investigate the structural relationship between the capsid structure of the ortho viruses (HIV, MLV, etc.), and the new structure of the foamy virus capsid [5] (PDB codes: 5m1g, 5m1h), the foamy virus structure was compared to one of the few full double domain ortho virus structures, the HIV capsid with PDB code: 3nte, using the flexible superposition program SAP [6]. Even though this program has a tolerant approach to relative domain shifts, the comparison produced a high RMSD value of 14Å over the 100 best superposed positions. The amino (N) terminal domain positions roughly corresponded but shifts in the relative orientation of the carboxy (C) terminal domain resulted in large deviations between equivalent helices. The superposed structures are shown in Fig. 1 a and the domain divergence can be seen clearly as a jump in the cumulative RMSD plot (Fig. 1 b).

### DALI searches

Although this initial superposition (Fig. 1) did not appear encouraging, the foamy virus structure was scanned across the Protein DataBank (PDB), using the DALI program [7] to search for any similarities.

#### Full chain scan

**Fig. 2:**
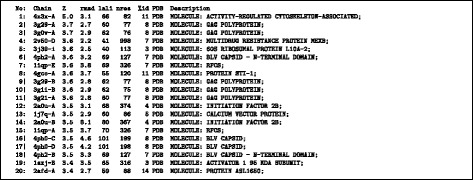
Top structural similarities. Found by the DALI program in the 90% non-redundant PDB (PDB-90) using the full length foamy virus capsid as a query (145 residues). The columns are: the ranked number of the hit (No.), marked by a ’|’ for a capsid protein, otherwise ’:’; the PDB entry identifier (Chain, with the chain designation after the dash); the DALI Z-score (Z) (significance estimate); the root-mean-square-deviation (rmsd) over aligned *α*-carbon positions; the number of aligned positions (lali); the number of residues in the matched structure (nres); the percentage sequence identity of the match (%id) followed by a description of the molecule. It can be seen from the number of matched positions (lali) that most matches are partial, covering typically less than half the query structure

**Fig. 3:**
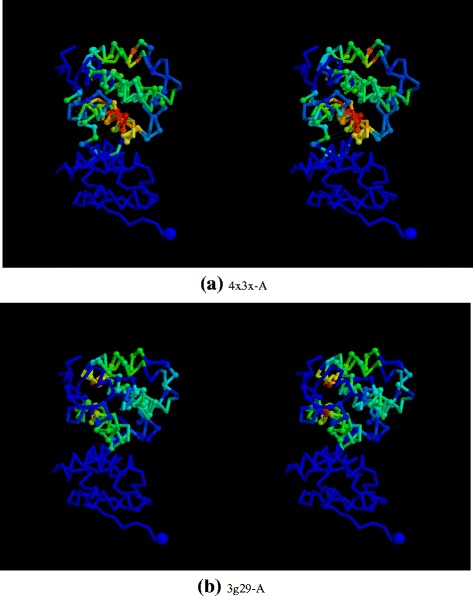
Top hits superposed. The top two DALI hits to the full foamy virus capsid are shown as a *α*-carbon backbone (stereo pair) coloured using the residue similarity score calculated by SAP. (*red* = strong similarity, *blue* = none). The amino terminus of the foamy structure is marked by a large ball and the other structure is distinguished by small balls on its *α*-carbon atoms. **a** a cytoskeleton associated protein (fragment) of the arc/arg3.1 gene (PDB code: 4x3x-A), (which is thought to have originated from a Ty3/Gypsy retrotransposon family capsid) and (**b**) the structure of the capsid C-terminal domain of the Rous scarcoma virus (PDB code: 3g29-A)

A scan of the full-length foamy structure using the DALI server^2^ over the 90% non-redundant protein structure databank identified a wide selection of retroviral capsid structures. In the ranked list of structure hits, capsids were identified from position 2 to position 550. The top hits are shown in Fig.2 (See Additional file 1 for a summary of the full 550 with Z-scores over 2). Many capsids are found in the top 20 hits and although the top scoring hit is not obviously a capsid protein, it is thought to have originated from the Ty3/Gypsy retrotransposon family *gag* gene [8]. However, almost all of these are partial hits, covering little more than half the query structure. The structural alignment of the top two hits is shown in Fig. 3 coloured to emphasise the matched regions.

The result of the DALI search indicated that the Foamy virus structure shares some similarity with the capsid structure of the ortho-viruses. However, the matches consist only of a small number of helices and appears barely more convincing than other matches to proteins that seem very unlikely to have any meaningful connection to a viral capsid. The preponderance of capsid matches throughout the list of hits might seem to add some support to the relationship but may simply be a reflection of the number of capsid structures in the structure databank.

Adding confusion to the ortho/foamy relationship is the additional observation that the distribution of matches to the ortho-virus structures between the amino (N) and carboxy (C) terminal domains are mixed. For example; taking the top 10 matches, the N-terminal domain of the Foamy structure aligns with 6 C-terminal domains and 4 N-terminal domains of the ortho virsuses and the best match with the corresponding Foamy C-terminal domain aligns with an ortho N-terminal domain.

#### Domain scans

**Fig. 4:**
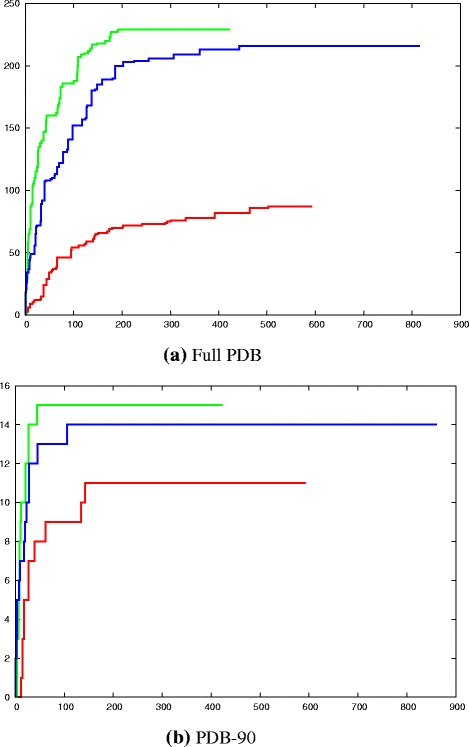
PDB capsid structure matches. The number of capsid structures identified by the DALI program in (**a**) the full PDB and (**b**) the 90% non-redundant PDB (PDB-90) is shown for queries using the full foamy capsid structure (*red*), the carboxy terminal domain (*green*) and the amino terminal domain (*blue*). The number of capsid hits (*Y*-axis) is plotted against the order of all hits ranked by Z-score down to a value of 2. A *curve* approaching the *top left corner* indicates greater specificity and the extent of a curve to the right indicates the total number of hits

To clarify the domain match specificity, the two domains of the Foamy virus (1–88 and 89–180, as defined automatically [9]) were scanned separately using the DALI program. The individual domains were much more specific at matching known capsid structures^3^, both in the full PDB and PDB-90 collections as can be seen from the plots in Fig. 4.

**Fig. 5:**
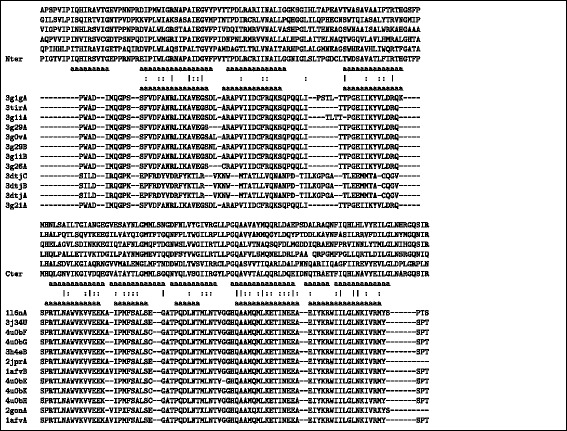
Top domain similarity alignments. The sequence alignments are shown for the top 12 capsid domain matches found by the DALI program using the foamy virus capsid N and C domains separately as a query over the full PDB. The sequence of the N-terminal domain (N-ter) is shown at the top of the first alignment block and the sequences of the C-terminal domain (C-ter) at the *top* of the second block. The sequences of the ortho-virsuses aligned below these all come from the “swapped” relationship of C and N terminal domains, respectively. These alignments, which are determined by structure not sequence, exhibit no specific similarity beyond what would be expected from aligning similar secondary structures from similar sized domains. (Amino acid identities are marked by a bar and similarities by a colon). The location of alpha helices is marked by the letter ‘a’, taken from the PDB entries of their adjacent proteins. A selection of other foamy virus sequences are aligned above the foamy virus sequence of known structure (human) which, from the *top*, are from: simian (orangutan), squirrel-monkey, cat, simian (unspecified) and horse. (NB. no alignment is implied between the two blocks of aligned domains)

The results of these scans strengthened the identification of the relationship to the ortho capsids and supported the swapped specificity for the N-terminal match of the Foamy structure with the C-terminal match of the ortho virus and *vica versa*, with all top 12 hits of each domain matching their opposed counterpart. The structure-based sequence alignments of each domain based on this equivalence are shown in Fig. 5.

Although domain transposition is not impossible in viral genomes, it is sufficiently unexpected to warrant deeper investigation, especially as it is hard to imagine how an ancestral capsid protein could tolerate such a large rearrangement and still pack to form a competent shell. We therefore undertook a more thorougher evaluation using alternative methods to assess the statistical significance of these structural similarities.

### Structural alignment significance

#### Reversed-structure searches

For each comparison, the DALI program calculates an empirical Z-score, combining an estimation of significance with protein length normalisation. The program reports all matches over Z=2, however, when the proteins are small and especially when the structures being compared are both predominantly alpha-helical in nature, then matches over this cutoff include many functionally unrelated hits where the similarity has arisen through the fortuitous alignment of a few helices.

**Fig. 6:**
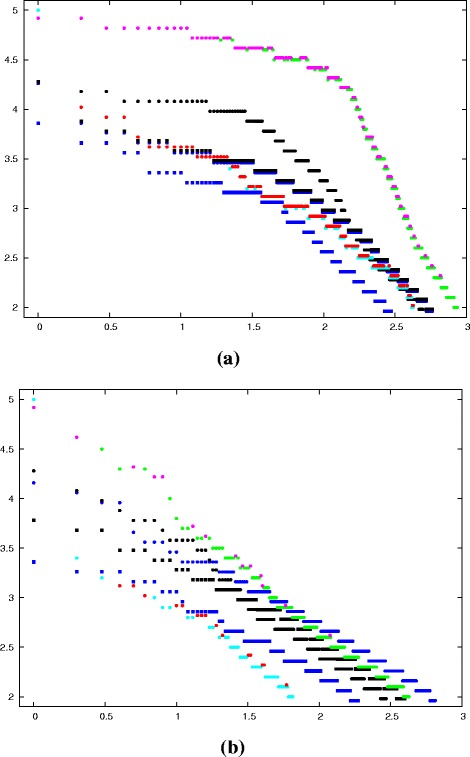
DALI scores with decoys. The DALI *Z*-scores (*Y*-axis) are plotted against the log _10_ of their ranked position in the list of hits (*X*-axis) with the amino-terminal domain (N) as T=*red*, F=*cyan* dots and the carboxy-terminal domain (C) as T=*magenta* and F=*green* dots, where T is a true capsid hit and F is a false hit to a non-capsid protein. Four sets of decoys are compared to these, consisting of the reversed foamy capsid domains in *black* and the reversed HIV capsid domains in *dark-blue* (with a circle = N and a square = C domains in both). The DALI score for each set of hits has been slightly displaced to prevent coincident dots from being obscured. (This happens because of the integral number of residues and the DALI score being specified to only one decimal place). **a** full PBD. **b** PDB-90

Therefore, to calculate a stricter cutoff on score, we created a decoy probe by reversing the alpha-carbon backbone then reconstructing the full atomic structure, using a simple algorithm to regenerate a full backbone^4^). Figure 6 plots the ranked DALI Z-scores for the separate (native) foamy domains. As would be expected, the larger C-terminal domain has hits with a higher significance than the smaller N-terminal domain: the former covers the range Z=2.5 to Z=5 over the true hits (magenta dots) whereas the latter tracks a similar profile running one Z-value unit lower (2–4 over true red dots). Plotting the Z-scores against the log of their rank produces almost linear traces for the hits from the PDB-90, making it easy to compare N-domain (red/cyan dots) with C-domain (magenta/green dots) (for T/F hits) in Fig. 6.

The equivalent scans with the reversed domain structures, using both the foamy and ortho (HIV) structures (neither of which should have any particular relationship to the capsid or any other natural protein) also found hits with high Z-scores (black and blue points in Fig. 6, respectively). When compared with the native domains (Fig. 6), these decoys had a profile that tracked mostly above the N-terminal native domain but below the C-terminal domain. However, with the latter domain, this was only distinct in the hits to the full PDB whereas with the PDB-90, the native domain was only clearly better over the top 10 matches, half of which were to non-capsid structures.

The results with the simple reversed decoy using DALI suggested that the match of the foamy virus domains to the ortho virus capsid N-terminal domain may be due to chance and that the match to the C-terminal domain looks meaningful if based on the hits to the full PDB but may be only marginal based on the PDB-90 hits.

**Fig. 7:**
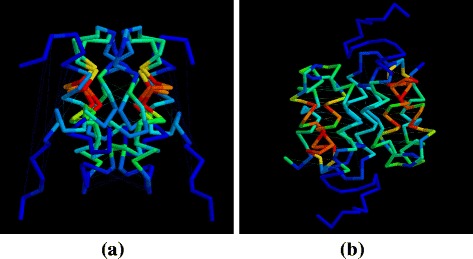
Native/decoy similarity. When superposed using the program SAP, both N-terminal (*left*) and C-terminal (*right*) domains have some degree of similarity to their reversed decoy ‘doppleganger’, which is more marked for the *N* domain. The superposed structures are coloured by the SAP residue-level score as *red* = high similarity, *blue* = low. The *N* domain has roughly 60 equivalent *α*-carbon positions compared to only 24 in the larger C domain. **a** N. **b** C

However, both the N and C terminal domains pocess a degree of internal symmetry which gives rise to a partial match with their reversed ’doppleganger’ decoys. The N-terminal domain superposed on its decoy had an RMSD of 5.4/60 (Å/ *α*-carbon s) and 5.5/24 for the C-terminal domain (Fig. 7). The higher symmetry of the smaller domain may be sufficient to explain its poor level of specificity seen in Fig. 6 and to try and resolve this ambiguity, a more diverse set of decoys was generated based on cyclic permutation and segment swapping combined with chain reversal [10].

#### Customised decoy comparisons

To improve the statistical analysis of the foamy/ortho capsid similarity, we employed a method based on the generation of a population of customised ‘decoy’ models to provide a background distribution of unrelated protein scores [10]. This method retains the advantage of the simple reversed structures where every comparison that constitutes the random pool is between two models of the same size and secondary structure composition as the pair of native structures being compared. For this study we collected 12 capsid N-terminal domains and 7 C-terminal domains, each of which were compared with the foamy N-terminal domain and the foamy C-terminal domain. (The structures are identified in Table 1 with full details in the “Methods” section).

**Table 1:** Ortho and foamy domain comparison Z-score statistics

*a*	ortho-N					
	foamy-N			foamy-C		
Virus	Pool	*a*-value	Z-score	Pool	*a*-value	Z-score
BLV6	300	0.552	**4.073**	244	0.542	3.692
BLV	251	0.550	**4.494**	184	0.400	3.669
HIV6	312	0.551	3.781	220	0.405	3.579
HIV1	312	0.573	3.703	213	0.402	3.692
HML2	264	0.777	2.166	196	0.438	**4.594**
HTLV	400	0.592	**4.030**	328	0.457	**4.013**
JSRV	225	1.063	0.896	190	0.601	3.237
MLV	326	0.751	3.044	188	0.508	3.151
MPMV	269	0.565	**3.902**	185	0.523	2.918
PSIV	285	0.621	3.731	235	0.369	**5.019**
RELIK	234	0.639	3.688	237	0.700	3.297
RSV	204	0.543	3.123	239	0.526	3.542
*b*	ortho-C					
BLV6	144	0.763	3.019	212	0.709	**4.046**
BLV	154	0.578	3.400	204	0.556	**4.047**
HIV1	157	0.593	3.760	174	0.705	3.362
HIV6	179	0.780	3.175	177	0.640	**4.380**
HML2	185	0.732	3.027	184	0.676	**3.900**
HTLV	156	0.685	3.847	163	0.694	2.807
RSV	155	0.448	3.754	235	0.403	**5.009**

For each amino (N) and carboxy (C) domain pair between an ortho virus structure and the foamy virus capsid structure, a **Z-score** is calculated based on the **a-value** (Equ ^*n*^. 1) derived from the comparison RMSD and length, relative to the **pool** of background decoy comparisons. The ortho **virus** identity is indicated by the code to the left, full details of which can be found in the “Methods” section. The top 12 Z-scores are high-lighted in bold, only three of which support a swapped domain match

**Fig. 8:**
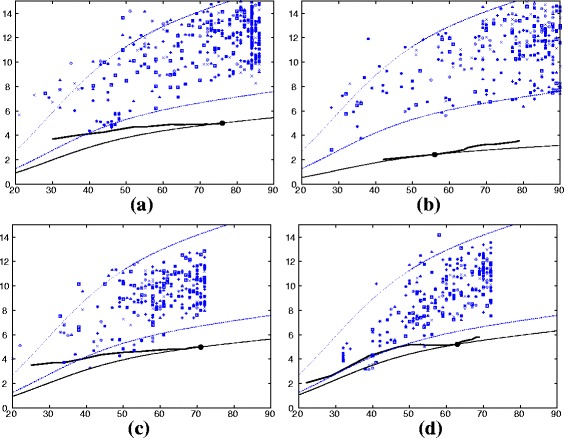
ortho/foamy domains compared with customised decoys. Each amino (N), carboxy (C) domain combination between the ortho retrovirus capsid structure (HIV1) and the foamy virus capsid structure is plotted as a line for increasingly large subsets of matched positions against their RMSD (*Y*-axis), as in Fig. 2. The point on this line marks the lowest *a*-value (Equ ^*n*^. 1), however, to be consistent with the decoy data, the full alignment length was used. The decoy comparison data (*blue*) is plotted in a variety of symbols with each representing a different combination of decoy construction. The *dashed blue lines* (which are the same in all plots) mark the approximate 10 ^*th*^ percentile boundaries of the decoy generated distributions, with a = 1.7 (*upper*) and a = 0.8 (*lower*). (See “Methods” section for details). **a** orthoN+foamyN. **b** orthoN+foamyC. **c** orthoN+foamyN. **d** orthoN+foamyC

For each domain pair to be compared, decoys were created using cyclic permutation and segment swapping with chain reversal to generate a family of customised decoys for each comparison [10]. All pairs of forward/reversed decoys were then compared, with each pair being drawn from a pool of models generated from the two native structures. This ensures that the native domains (which may have different lengths) are always evaluated against a decoy pair with the same length combination. (See Methods section for details). All the decoy comparisons, of which there are typically 150–300 for each comparison, can then be compared to the native pair on a plot of RMSD against the number of matched residues (*α*-carbon atoms). An example is shown in Fig. 8(c) for the comparison of the HIV1 structure (PDB codes: 1ak4 (N) and 1a43 (C)) domains against the foamy virus Gag domains.

#### Statistical analysis of the decoy comparisons

The quality of the comparisons in Fig. 8 c can be quantified as a combination of their RMSD (*R*) and the number of matched (superposed) positions (*N*). However, as explained in the “Methods” section, for statistical analysis, it is easier to combine this pair of numbers as a single number, called the *a*-value (Equ ^*n*^. 1), which is the scaling factor that causes a theoretical curve to pass through the point (*R*,*N*).

**Fig. 9:**
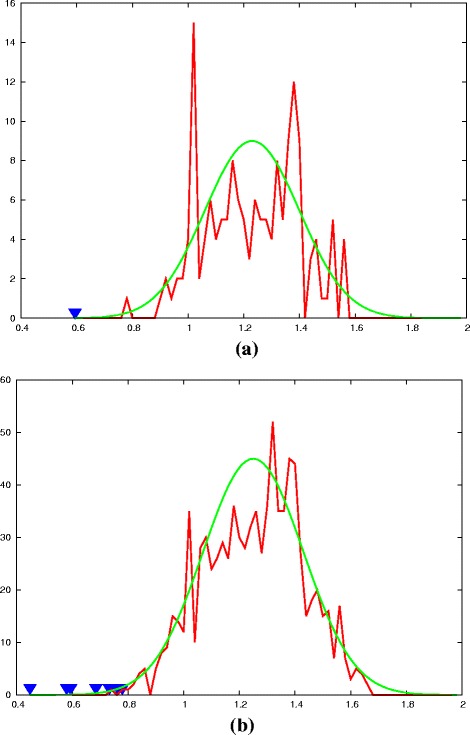
ortho-C and foamy-N domain comparison statistics. The *a*-value (normalised RMSD) for the comparison of the ortho-C and foamy-N decoy domains (Fig. 8 b) are plotted as a frequency distribution (*red*) along with a bell-shaped Normal distribution curve (*green*) with matching mean (*μ*) and spread (*σ*). Part (**a**) shows the distribution for the HIV1 C-terminal domain (*μ*=1.23) and spread (*σ*=0.17) with the position of the native structure comparison plotted as a *blue* (inverted) *triangle*. Its position lies 0.64 units below the mean giving a Z-score of 0.64/0.17=3.76. Part (**b**) shows the combined data from seven representative viruses (in Table 1). These data comprise two distributions, that of the combined decoys and also the much smaller distribution of native scores (*blue triangles*). This allows a T-test to be made on the significance of their separation

When expressed by a single *a*-value all the data points in a comparison, such as Fig. 8 c, can be plotted as a frequency histogram and examined to see if they approximate a Normal distribution. The distributions were found to be a good fit to unskewed Gaussians and so were treated as normal distributions (rather than extreme value distributions that have also been considered previously as a model for random structure comparison scores [10, 11]). The frequency data from the comparison of the orthoN domain from HIV1 and the foamyC domain (Fig. 8 c) is shown in Fig. 9 a along with a Normal distribution that has the same mean (*μ*) and standard deviation (*σ*) as the data. On this plot, the value of *a* (Equ ^*n*^. 1) for the comparison of the native pair of domains is also plotted (blue triangle) and from its position, a Z-score can be calculated.

In this way, the significance of all combinations of the native ortho and foamy domain superpositions were calculated, using the background distribution of ‘customised’ decoy comparisons based on each individual native pair. The resulting Z-scores (*σ* units) are collected in Table 1. The degree of similarity between the domains ranged from less than 1 *σ* to over 5 *σ*, with the latter (highly significant) result being obtained for both a swapped (NC) and forward (CC) combination. However, of the top 12 scores, only three now came from swapped pairings.

##### Asymmetry statistics:

**Fig. 10:**
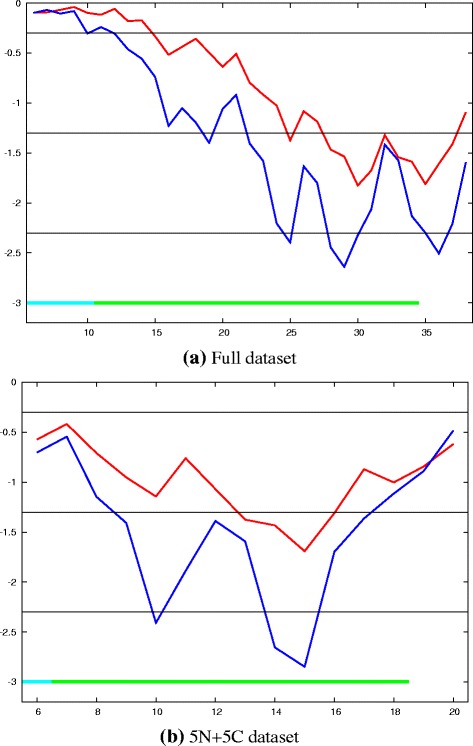
Asymmetry statistics for like/mixed domain pairs. Given the ranked list of domain pairings, the chance for more domain pairs of like-type to be found higher than the observed order was evaluated from empirical distributions measured by two statistics: the second moment of the rank value (*red*) and the number of like-type pairs in the top half (*blue*). These statistics were calculated for all subsets from the 6 top pairs up to the full set of comparisons (*X*-axis) and for each, the chance of a better score is plotted as the log _10_ of the probability (*Y*-axis). The *horizontal lines* mark the 0.5, 0.05 and 0.005 levels. The line at the 0.001 level is coloured by the Z-score for each pair as: *green* = over 3 and *cyan* = over 4 sigma. Part (**a**) shows the probabilities calculated from the full set of 7 carboxy and 12 amino domains and part (**b**) shows the same values calculated on a more balanced set of 5 non-redundant carboxy domains and their matching amino domains

To quantify the degree of bias for domains of like-type (NN, CC) to be more similar than those of mixed-type (NC, CN), the observed ranking of like and mixed pairs, based on their Z-value (Table 1), was compared to that expected by chance. The positions of all pairs in the list were shuffled a million times and the asymmetry of each arrangement was quantified as the number of like-pairs in the top half and also by their second moment: $\surd \left (\left (\sum r^{2}_{i}\right)/N\right)$, where *r* is the rank of the like-pair *i* in a list of *N* pairs. The chance of obtaining a distribution with more like-pairs being ranked higher can be caluclated by summing the area of the tail of each empirical distribution that lies beyond the observed value. However, these values were calculated over all pairs and neglects the principle that emphasis should be given to the more significant similarities. Rather than rely on a single significance cutoff (like 3 *σ*) or an arbitrary cutoff (like the “3-out-of-12” mentioned above), we calculated statistics for all such cutoffs (Fig. 10 a).

The majority of values in Fig. 10 a lie below the 0.05 probability level for the larger sample sizes, with those for the top-half bias statistic (blue line) being more significant than the moment-based statistic (red line). While confirming the visual trend towards a bias of higher scoring like-type domain similarities, the analysis summarised in Fig. 10 a is complicated by having unequal numbers of amino and carboxy domain comparisons and also by including some closely related structures. To produce a more balanced data-set, one of each pair of the two most similar carboxy domain structures was discarded leaving five structures and for each of these, their matching amino terminal domain was also retained, leaving: BLV-1, HIV-1, HML2, HTLV-1 and RSV. Despite having a smaller set of comparisons (5N + 5C domains giving 20 rather than 38 Z-scores), the results for this reduced set indicated an equally clear bias towards towards a preferred like-domain equivalance, especially as measured by their occurrence in the upper half of the ranked list, with several having a probability below the 0.05 level and a few below the 0.005 level (Fig. 10 b).

##### T-test statistic:

An alternative to the above analysis, which still remains marginally significant, is to pool the raw comparison data for all the domain comparisons and their background distributions giving now not just a single value compared to a distribution but two distributions (Fig. 9 b). For these data, a significance was calculated using Student’s T-test, the values of which are given in Table 2.

**Table 2:** ortho and foamy capsid domain comparison T-test significance

	orthoN	orthoC
foamyN	Avg: 6.67e-01 < 1.32e+00	Avg: 6.51e-01 < 1.25e+00
	Tprob = 4.62e-21 **	Tprob = 2.35e-16 **
	StD: 1.61e-01 = 2.12e-01	StD: 1.17e-01 = 1.89e-01
	Fprob = 1.84e-01	Fprob = 1.12e-01
foamyC	Avg: 4.92e-01 < 1.29e+00	Avg: 6.22e-01 < 1.30e+00
	Tprob = 4.09e-10 **	Tprob = 3.81e-23 **
	StD: 1.02e-01 < 2.21e-01	StD: 1.12e-01 = 1.77e-01
	Fprob = 7.37e-03 **	Fprob = 1.20e-01

For each combination of domains between the ortho and foamy viruses, the probability is given that the two means from each distribution (Avg values) were sampled from the same distribution. (i.e., that the native and decoy comparisons are not distinct). All domain pairings are extremely significant. An F-test was used to test if the standard deviations (Std) of each sample were distinct and if not, the a T-test was made on the assumption of equal standard deviations

From these results, it can be seen that all the four possible pairings are highly significant with probabilities ranging from 10^−10^ to over 10^−20^. It is also clear that the two swapped pairings (NC and CN) have higher probabilities than the forward pairings (NN and CC). Combining the probabilities (*P*) as: *Δ* *P*= log10(*P*_*NN*_ *P*_*CC*_)−*l* *o* *g*_10_(*P*_*NC*_ *P*_*CN*_), gives a value of 17.7 (42.7 - 25.0) which means that the swapped pairing is almost 18 orders of magnitude less likely than the forward pairing. Calculating the same statistic on the reduced 5N+5C domain data set gave a similar result but with a difference reduced 1000-fold to 15 orders of magnitude.

The unexpected swapped pairing, which was indicated originally by the DALI results, now seems less likely. The preferred, and biologically more reasonable, result is that the ortho virus domain are related to the foamy virus domains as a result of genetic divergence from a common, double domain ancestor.

### Internal duplication

**Fig. 11:**
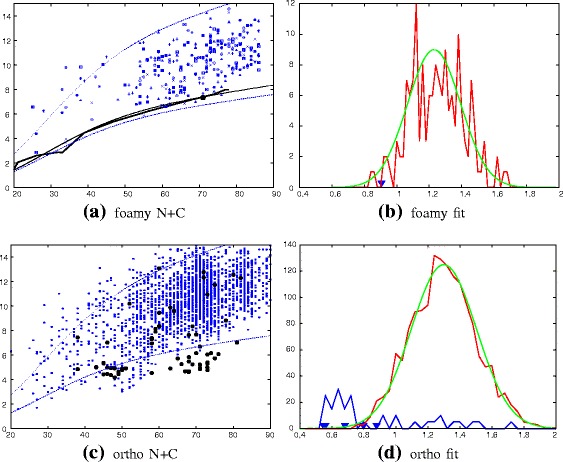
N and C domains compared with customised decoys. **a** The N and C domains of the foamy virus (*black*) compared to decoys (*blue*) with (**b**) the derived frequency plot with the native comparison marked by a *blue triangle*. (See legend to Fig. 8 for details). **c** The N and C domains of the ortho virus combinations (*black*) with (**d**) the derived frequency plot showing the native comparison for pairs from the same virus (*blue triangles*) with the distribution of all native pairs shown as a scattered frequency plot (*blue line*). (See “Methods” section for details)

The transposed pairings of N/C and C/N (ortho/foamy) domains still retain a high structural significance and this suggests that the two domains are derived from a common ancestral structure, probably as the result of a prior gene-duplication event that has been retained more clearly in the less embellished foamy virus structures. Comparing the two foamy domains gives a Z-score of 2.077 sigma which, although of marginal significance, supports this model. (Fig. 11 a, b).

Such a relationship between the foamy domains implies an equivalent relationship in the ortho viruses and a similar comparison in structures of their N and C domains finds matches with Z-scores ranging from 2 to 4. As with the comparison of the ortho and foamy structures, these can be pooled to allow a joint T-test to be applied. This gave a probability of 10^−8^ that the true N/C domain comparisons were drawn from the decoy distribution, adding strong support to the hypothesis of an ancient gene duplication occurring before the split of the ortho and foamy virus families. (Fig. 11 a, b, *blue triangles*). Supporting this relationship, earlier studies also suggested an internal duplication in the ortho virsuses but were based largely on very distant sequence similarity [12].

This test was applied only to the comparison of domains between viruses with known structures for both domains, however, it is not unreasonable to compare amino and carboxy domains across all viruses. The longer loops in the ortho virus domains gives greater scope of structural variation and a wide range of variation was seen ranging from RMSD values under 4 to over 12. When normalised for length (*a*-value from Equ ^*n*^. 1) and partial matches under 60 positions excluded, a distinct cluster remains between *a*=0.5…0.8 (4...6Å RMSD) but still with a long tail to higher values. Despite this tail, the T-test on the distributions is highly significant at 2.7×10^−17^.

**Fig. 12:**
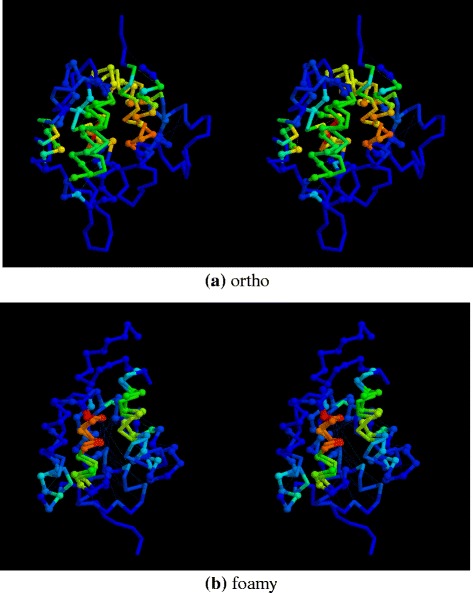
Amino and carboxy domains superposed. **a** ortho virus domains and (**b**) foamy virus domains are shown as a stereo pair with their *α*-carbon backbones coloured by the residue similarity score calculated by SAP. (*red* = strong similarity, *blue* = none). The amino terminal domain is distinguished by small balls on its *α*-carbon positions and the amino terminus lies to the top in both panels

One of the better N/C ortho similarities is shown in Fig.12 a, along with the N/C ortho domain superposition in Fig.12 b.

### Fold-space representation

To summarise the structural relationships among the ortho and foamy domains, the matrix of pairwise comparisons was projected into a three-dimensional fold-space. (See “Methods” for details). This produces a best visual representation of the RMSD values between domains.

**Fig. 13:**
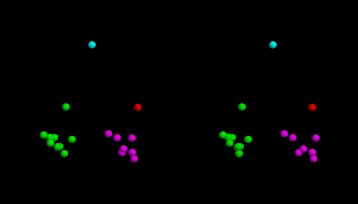
Fold-space representation of all domains. All the viral domains considered in the paper were projected into a 3D fold-space representing the relationship of their SAP weighted RMSD values. The domains are coloured as: foamyN = *cyan*, foamyC = *red*, orthoN = *green* and ortho C = *magenta*

As can be seen from Fig. 13, the N and C domains of the ortho viruses form distinct clusters with the foamy C domain lying closer to the ortho C-domain cluster. The foamy N-domain, however, maintains a fairly equal distance from both ortho domain clusters but lies closer to its C-terminal partner.

## Discussion and conclusions

### Structure comparison

#### Pairwise significance

The comparison of small domains that are largely composed of *α*-helices presents a challenging problem in how to interpret the significance of the RMSD values. As the individual helical secondary structure elements (SSEs) constitute a sizeable fraction of the domain, it takes only the chance alignment of a few helices to result in a low RMSD over a large proportion of the structure, giving an apparently meaingful result.

The use of the customised decoy-model sets, as illustrated here, attempts to avoid this problem by recreating a large number of possible folds that were generated using the same (reconnected) SSEs. Moreover, to avoid any chance recreation of native fragments, each comparison always involved the comparison of a native (forward) chain direction with a reversed chain. Using these models, a background distribution of decoy/decoy comparisons allowed us to calculate Z-scores for each native/native comparison between the different Gag proteins. This has the advantage that every comparison in the background distribution involved two models with the same length, residue packing density and secondary composition as the native pair. These values indicated a clearly significant relationship between the foamy and ortho CA structures.

#### Direct or transposed domain order?

Although the decoy model alignment strategy did confirm the relationship between the foamy and ortho CA structures, the Z-scores did not point to a clear resolution of whether the domains should have a direct correspondence (NN and CC match) or a transposed relationship (NC and CN) as significant individual matches were found across all pairings. Testing for a bias towards more significant like-domain pairings (NN, CC) in the list of similarities ranked by Z-score confirmed the visual bias towards a natural correspondence but only at a marginal level of significance (around 0.05). By contrast, the application of a T-test on the combined raw comparison data returned a very clear distinction between the direct and the transposed relationships, clearly favouring the more natural forward order.

However, although the “astronomic” probabilities calculated by the T-test seem very convincing, they must be viewed in the light of the much lower probabilities calculated from the asymmetry statistics. Both calculations involve assumptions and are limited by the small number of known structures so neither can be taken as definitive. Nevertheless it would seem likely that the “true” level of significance may lie somewhere between the two results and as both of these objective assessments point in the direction of the NN and CC domain order, there is no reason to adopt the more unexpected transposed domain order.

### Evolutionary implications

On the basis of these structural comparisons, and a variety of recently described functional assays [5], we can conclude that the central region of the spumavirus *gag* gene encodes a polypeptide sequence related to that of the corresponding region of orthoretroviral, CA. It therefore seems reasonable to suppose that the last common ancestor of orthoretroviruses and spumaviruses possessed such a sequence. Moreover this region appears to be made up from two related all helical subdomains suggesting a gene duplication event in a common precursor.

In our initial search employing foamy virus CA using the DALI program, we made the observation that the strongest similarity of the foamy virus CA domains was actually with a cellular protein, Arc (Activity-Regulated Cytoskeleton-associated protein). Arc is required for neural synaptic growth and activity [13–16] and mis-regulation and/or deletion contributes to diseases of cognition [14, 16, 17]. Arc has widespread and clear sequence homologues as far back as insects and probably deeper, giving it a very ancient origin somewhere close to the metazoan root [12, 18] and based on sequence homology Arc is considered to be a relic of an ancient Ty3/Gypsy retrotransposon [8], preserved as a ‘living fossil’ in metazoan genomes. Given the structural relatedness of foamy virus CA and Arc, this might suggest an equally ancient origin for foamy virus CA. As it is believed that the Ty3/Gypsy family of retrotransposons gave rise to retroviruses [19], it will therefore be of considerable interest to determine whether the Gag of Ty elements also comprise CA proteins with a two-domain structure.

It is also noteworthy that Ty3 Gag is significantly smaller than that of the foamy and orthoretroviruses and although it contains CA related sequences there is no equivalent of either orthoretroviral MA or PFV Gag-NtD, regions of Gag necessary for membrane targeting, budding and extracellular release of virions. Therefore, given the very different structures of MA [20–23] and Gag-NtD [4], this raises the possibility that the MA and Gag-NtD domains of the orthoretroviruses and foamy viruses were co-opted by independent events that has resulted in the viruses employing different mechanisms to facilitate budding from the cell. Notably, Gag from Gypsy, an Errantivirus capable of extracellular replication [24] and Arc contain additional N-terminal domains. In Gypsy-Gag this domain is distantly sequence-related to orthoretroviral MA [12]. By contrast, in Arc it contains a coiled coil region [8] reminiscent of spumavirus Gag-NtD [4, 25] further supporting the notion of a shared origin for Arc and foamy virus Gag that is distinguishable from an alternative acquisition pathway giving rise to Gypsy and the orthoretroviruses.

## Methods

### Structural data

The foamy virus structures were obtained from the Protein Structure Databank (PDB code:5M1G) [5].

The ortho virus structures used, with their shorthand code in bold and PDB code in teletype, were:

- **BLV**: bovine leukemia virus (deltaretrovirus) 4PH1 (N-ter.dom) and 4PH2 (C-ter.dom) [26],
- **BLV6**: bovine leukemia virus (hexameric) 4PH0 (both dom.s) [26],
- **HIV1**: human immunodeficiency virus 1 (lentivirus) 1AK4 (N-ter.dom) [27] and 1A43 (C-ter.dom) [28],
- **HIV6**: human immunodeficiency virus 1 3H47 (both dom.s) [29],
- **HML2**: human endogenous retrovirus type-K (betaretrovirus) [30],
- **HTLV**: human T-cell leukemia virus (deltaretrovirus) 1QRJ (both dom.s) [31],
- **JSRV**: jaagsiekte sheep Retrovirus (betaretrovirus) 2V4X (N-ter.dom) [32],
- **MLV**: murine leukemia virus (gammaretrovirus) 1U7K (N-ter.dom) [33],
- **MPMV**: Mason-Pfizer monkey virus (betaretrovirus) 2KGF (N-ter.dom) [34],
- **PSIV**: prosimian immunodefficiency virus (ancient lentivirus) 2XGV (N-ter.dom) [35],
- **RELIK**: rabbit endogenous lentivirus type-K (ancient lentivirus) 2XGU (N-ter.dom) [35],
- **RSV**: Rous sarcoma virus (alpharetrovirus) 3G1I (both dom.s) [36].

### Structure comparison

#### DALI

The DALI method for searching the PDB with a structural query [7] was accesed via the server at: http://ekhidna.biocenter.helsinki.fi/ dali_server. The DALI method reports the significance of each match with an estimated Z-score which is the raw comparison score, normalised by the combined length of the proteins. Z-scores down to a value of 2 are reported by the program.

The list of DALI hits (ranked by Z-score) were assessed by how many high-scoring capsid structures had been identified. These true/false (T/F) hits were defined simply by protein descriptions that contained the words “CAPSID”, “GAG” or “P24”. This may have misclassified a few (low scoring) hits to the matrix protein and missed some hits where the primary description refers to a cyclophilin structure solved in complex with the capsid.

DALI reports structural hits in both the full PDB and a reduced collection of structures that have no pair of proteins with over 90% sequence identity, referred to as the 90% non-redundant or PDB-90 collection. It was found, however, that some hits, seen in the full PDB were not found in the PDB-90, for example in Fig. 6, all of the top 31 hits of the N-domain against the full PDB are missing in the PDB-90 hits. The most likely explanation is that the PDB-90 secection has not been updated at the same time as the full collection. For this reason, hits to both databases were monitored.

#### SAP

The SAP method for structure comparison [6] was run as a local copy which can be accessed at: https://github.com/WillieTaylor/util. As part of determining the alignment between two structures, the SAP program calculates a similarity score for each pair of matched positions which is how similar the rest of the structure looks from the viewing-frame of the superposed residues. This value can be used both to weight the importance of positions when calculating the (rigid-body) RMSD superposition and to colour positions in the superposed structures [37]. (As in Fig. 3).

If the matched positions are ranked by this value, then RMSD values can be calculated over increasingly larger subsets to high-light the extent of a well matched core before the contribution of variable loops, or domain shifts, leads to higher RMSD values. (As in Fig. 1 b).

### Decoy structure construction

#### Reversed structure decoys

Simple structural decoys were generated from native PDB structures by reversing the order of the *α*-carbon atoms in the PDB file using the Unix command line:

cat native.pdb | grep ’ CA ’ | sort -nr -k2 > reverse.pdb

The reversal of a protein chain does not alter the chirality of the alpha helix and these decoys can be used directly in SAP. However, DALI requires all main-chain atoms and these must be regenerated for the reversed decoys. This was done using the simple ca2main program which can also be found at: https://github.com/WillieTaylor/util. The method is based on the geometry of the *α*-carbon-virtual chain using relationships described in ref. [38].

#### Customised decoys

Customised structural decoys were generated for each comparison using each of the pair of structures being compared to create two pools of decoys then comparing all decoys in the first pool against all decoys from the second but with their chain reversed as described in the previous section.

The decoys were created as described in Ref. [10]: starting by cyclising the chain then introducing new termini in each surface loop to create cyclic permutations. In addition, when three loop regions lie in close proximity, their ends are also reconnected in such a way that if a chain, comprising four segments (1…4) runs from amino (N) to carboxy (C) termini through three adjacent loop regions a-b, c-d and e-f (i.e.: N,1,a-b,2,c-d,3,e-f,4,C) then the reconnected chain runs: N,1,a-d,3,e-b,2,c-f,4,C with each switch being made at the least disruptive point between a pair of loops. This chain switching does not create any reversed segments which would otherwise form regions of local matching when the whole chain is reversed.

In a pair of structures, if each have four surface loops where breaks can be made, then including the native termini, this gives five cyclic permutations and if two groups of loops can be reconnected then a total of 15 distinct decoys can be made from each native starting structure. As these can be compared pairwise, a pool of 225 decoy derived data points is generated that constitutes the random background against which the native/native comparison can be assessed.

For example, in Fig. 8, the 36 data points marked by a solid circle come from the comparison of six cyclic permutations of a native ortho domain compared with six permutations of a reversed foamy domain that includes a single loop reconnection.

Every pair drawn from this pool will have the same lengths as the two native structures as well as the same secondary structure composition, surface exposure, residue packing density and inertial properties but each decoy will have a different chain fold.

### Statistical tests

#### RMSD length normalisation

The quality of structure comparisons can be characterised by a combination of their RMSD value and the number of matched (superposed) positions. How to combine these values has been the subject of much discussion over the years and central to this is the expected random RMSD value for two proteins of a given length [39–41]. However, when reviewed [10], all these measures were approximations of a simple square-root function of the protein length (as originally proposed by McLachlan on theoretical grounds [39]) but with an added term to depress the RMSD values obtained with small units or structure that are dominated by secondary structure elements (and super-secondary structure motifs) giving a lower than expected RMSD value. The formula that best captures this is: *R*=*√* *N*(1− exp(−*N* ^2^/*s* ^2^)), where, *R* is the expected random RMSD for *N* matched positions and *s* is the damping factor in the inverted Gaussian term (equivalent to the standard deviation in the Normal distribution).

Any point that lies on this line can be considered “exactly” random with those above it being “more” random and those below it “less” random. This can be quantified as a single number which is the value of a scaling factor (*a*), which when applied to the curve, makes it pass through any given point. If a comparison has an RMSD of *R* over *N* positions, then *R*=*a* *√* *N*(1− exp(−*N* ^2^/*s* ^2^)) and when

1 $$  a = R/(\surd N (1-\exp(-N^{2}/s^{2}))),  $$

the line will pass through the data point. This reduces the pair of values (*R*,*N*) to a single value *a* that is a simpler quantity for statistical analysis.

The best value for *s* is slightly dependent on the nature of the proteins being compared. For artifical (random-walk) models with no secondary structure, no modification will be needed but the proteins considered here have segments of packed alpha helices that can be locally similar over two to three helices. To correct for this, a value of *s*=30 was used (or 1/*s* ^2^=0.11) which is higher than the value of 1/*s* ^2^=0.03 used previously. That this is a reasonable fit to the data can be seen in the way the dashed blue lines in Fig. 8 track the upper and lower boundary of the decoy comparison results.

When *a*=1, the point lies on the random line and when *a*=0, the RMSD is zero, so values of *a* that approach this lower bound will be of interest when evaluating similarity.

#### Frequency plots

The *a*-values obtained using Equ ^*n*^. 1 were plotted as frequency histograms using using only data points that had a length of *N*±10, where *N* is the maximum number of matched positions in the comparison of the two native structures. As the sample size is small (typically, 100–300), these plots are quite noisy but their overall distribution does not deviate too greatly from a Gaussian distribution. This was tested on the difference between the observed and ideal cummulative distribution functions (CDFs) using the Kolmogorov-Smirnov test in the statistical package "R". Of the 38 samples from each domain pairing, the null hypothes "that the sample was drawn from a Normal distribution" could be rejected in only two cases with a confidence below the 0.01 significance level or three below the 0.05 level. (See the Additional file 1 for details). The underlying distribution becomes more apparent when the data sets are combined in Fig. 11 d.

Previously, a cumulative plot of RMSD was used to select an optimal value for *N* (giving the minimum *a*-value). This can be important if the full set of matched positions is dominated by a high deviations from variable loop regions. However, in the current application, the small length of the foamy virus loops meant that this was not an important aspect and the full number of matched positions was taken. Otherwise, the same correction would have to be applied to all decoy comparisons to maintain a fair comparison. (See Fig. 8, where the black dot marks the minimum *a*-value length).

The mean and standard deviation of the *a*-values in the *N*±10 region were calculated and the corresponding Normal distribution used to calculate Z-scores for the associated native comparison. (See Fig. 9 a, for an example).

#### T-tests

Data from separate native/native comparisons, with their customised decoy data, were combined giving not only a much larger background population of decoy derived scores but also a small population of native comparison scores that can be tested to calculate the probability that they were drawn from the same population as the decoy data. To do this, a T-test was used which takes the size, mean, and standard deviation of each distribution and calculates a probability. The implementaion of this test was taken from the Numerical Reicpies collection [42] which implements one of two variants of the test depending on whether the distributions have statistically distinct standard deviations. (Routines ttest() and tutest()). The choice of routine is based on a preapplication of an F-test on the standard-deviations. (Using the routine ftest()).

The values quoted in the Results section are for a two-tailed T-test, however, as it is expected that the native comparisons should always be more similar than comparisons between random models, then a one-tailed T-test would be valid, which gives half the probability. As the values in the Tables are so significant and only the relative relationships are of interest, then the choice is unimportant.

### Fold-space clustering

The results of the pairwise similarity within a set of structures can be visualised by treating the RMSD values as Euclidean distances^5^ and reducing their dimensionality to sufficiently few dimensions to be visualised: usually 2D or, better 3D, to visualise the space with less distortion. Rather than use a simple multi-dimensional scaling (MDS) method ([43]), the more complicated method of multi-dimensional projection was used ([44], see [45] for a simpler exposition).

This method reduces the dimensionality of the projection in gradual stages with each step employing triangle-inequality balancing and hyper-dimensional real-space refinement. In the real-space refinement stages, a weight can be applied to pairwise distances. (This cannot be done in direct MDS projection, which can only assign a mass to each point). Weights were assigned to distances as a function of their inverse RMSD, up to a maximum value of 1.

The method is robust and has been widely applied to rough models ([46]) and predicted inter-residue distances that constitute highly non-metric data sets ([47]).

## Endnotes

^1^ This class is also commonly referred to as the Foamy viruses (after the morphological effect they have on infected cells) and will be referred by this name frequently below, with the term orthoretroviruses also contracted to “Ortho viruses”.

^2^ http://ekhidna.biocenter.helsinki.fi/ dali_server, see “Methods” section for details.

^3^ True/false hits were defined by protein descriptions with the words “CAPSID”, “GAG” or “P24”.

^4^ Note that reversing the *α*-carbon backbone does not change the chirality of the *α*helices but as DALI requires a full atomic backbone, this must be restored on the reversed chain.

^5^ In theory, pairwise RMSD values are guaranteed to constitute a consistent Euclidean metric, but only in N-1 dimensions (where N is the number of structures compared).

## Additional file

Additional file 1 Supplementary Material. (PDF 40 kb)
