# Supplementary material for: A comparative analysis of the foamy and ortho virus capsid structures reveals an ancient domain duplication
Source: BMC Struct Biol. 2017 Apr 4;17:3. doi: 10.1186/s12900-017-0073-0 (PMC5379526; doi:10.1186/s12900-017-0073-0)
Supplement: Supplementary file 1 — Supplementary Material. (PDF 40 kb) [file 12900_2017_73_MOESM1_ESM.pdf]

Supplementary Material

Normal distribution test

The distributions of the a-statistic (Equ^{n}. 1) generated from each pairing of ortho and foamy virus domains was tested for Normality using the Kolmorgorov-Smirnov (KS) test using its implementation in the statistical package "R" (https://cran.r-project.org/doc/manuals/r-release/R-intro.pdf). In the following list of results, only two distributions were found to deviate significantly (below the 0.01 level, dashed line) from the null hypothesis that the distributions are Normally distributed.

```
Virus domains   length  KS D value  p-value

HTLV_C + foamyN   73 D = 0.0426, p-value = 0.9636
HTLV_N + foamyN   77 D = 0.0261, p-value = 0.9495
HIV6_N + foamyN   77 D = 0.0301, p-value = 0.9367
HML2_N + foamyC   79 D = 0.0469, p-value = 0.8327
BLV_N + foamyN    79 D = 0.0397, p-value = 0.8287
HIV1_C + foamyC   69 D = 0.0492, p-value = 0.8254
HML2_C + foamyN   72 D = 0.0545, p-value = 0.7042
HIV6_C + foamyC   60 D = 0.0599, p-value = 0.5758
BLV6_N + foamyN   79 D = 0.0462, p-value = 0.5727
HIV6_N + foamyC   80 D = 0.0576, p-value = 0.5603
RSV_N + foamyC    79 D = 0.0533, p-value = 0.5451
RELIK_N + foamyC  87 D = 0.0526, p-value = 0.5441
BLV_N + foamyC    80 D = 0.0656, p-value = 0.4732
HIV1_N + foamyN   76 D = 0.0537, p-value = 0.4342
BLV6_C + foamyC   72 D = 0.0632, p-value = 0.3804
RELIK_N + foamyN  78 D = 0.0605, p-value = 0.367
HIV6_C + foamyN   59 D = 0.0695, p-value = 0.3497
BLV6_C + foamyN   74 D = 0.0865, p-value = 0.2894
HTLV_C + foamyC   72 D = 0.0841, p-value = 0.2643
MLV_N + foamyC    80 D = 0.0769, p-value = 0.2638
JSRV_N + foamyC   81 D = 0.0774, p-value = 0.2547
HIV1_C + foamyN   71 D = 0.086, p-value = 0.2479
RSV_N + foamyN    72 D = 0.0815, p-value = 0.2295
MPMV_N + foamyC   80 D = 0.0826, p-value = 0.1884
HIV1_N + foamyC   79 D = 0.0794, p-value = 0.1867
JSRV_N + foamyN   78 D = 0.0737, p-value = 0.1796
PSIV_N + foamyN   79 D = 0.0685, p-value = 0.143
BLV6_N + foamyC   79 D = 0.0769, p-value = 0.1376
MPMV_N + foamyN   78 D = 0.0713, p-value = 0.1321
BLV_C + foamyC    66 D = 0.0881, p-value = 0.09677
HML2_C + foamyC   80 D = 0.0956, p-value = 0.09418
PSIV_N + foamyC   80 D = 0.09, p-value = 0.06448
BLV_C + foamyN    70 D = 0.1083, p-value = 0.06359
HML2_N + foamyN   76 D = 0.0886, p-value = 0.06022
```

```
MLV_N + foamyN    80 D = 0.0751, p-value = 0.05412
RSV_C + foamyN    72 D = 0.1244, p-value = 0.02049
-------------------------------------------- 0.01
HTLV_N + foamyC   82 D = 0.1014, p-value = 0.008952
RSV_C + foamyC    69 D = 0.1408, p-value = 0.000203
```

DALI hits

Matches to PDB90

No:	Chain	Z	rmsd	lali	nres	%id	PDB	Description
1:	4x3x-A	5.0	3.1	66	82	11	PDB	MOLECULE: ACTIVITY-REGULATED CYTOSKELETON-ASS
2|	3g29-A	3.7	2.7	60	77	8	PDB	MOLECULE: GAG POLYPROTEIN;
3|	3g0v-A	3.7	2.9	62	76	8	PDB	MOLECULE: GAG POLYPROTEIN;
4:	2v50-D	3.6	2.2	41	998	7	PDB	MOLECULE: MULTIDRUG RESISTANCE PROTEIN MEXB;
5:	3j39-i	3.6	2.5	40	113	3	PDB	MOLECULE: 60S RIBOSOMAL PROTEIN L10A-2;
6|	4ph2-A	3.6	3.2	69	127	7	PDB	MOLECULE: BLV CAPSID - N-TERMINAL DOMAIN;
7:	1iqp-E	3.6	3.8	69	326	7	PDB	MOLECULE: RFCS;
8:	4gco-A	3.6	3.7	55	120	11	PDB	MOLECULE: PROTEIN STI-1;
9|	3g29-B	3.6	2.8	62	77	8	PDB	MOLECULE: GAG POLYPROTEIN;
10|	3g1i-B	3.6	2.9	62	75	8	PDB	MOLECULE: GAG POLYPROTEIN;
11|	3g21-A	3.6	2.8	60	77	8	PDB	MOLECULE: GAG POLYPROTEIN;
12:	2a0u-A	3.5	3.1	68	374	4	PDB	MOLECULE: INITIATION FACTOR 2B;
13:	1j7q-A	3.5	2.9	60	86	5	PDB	MOLECULE: CALCIUM VECTOR PROTEIN;
14:	2a0u-B	3.5	8.1	80	367	4	PDB	MOLECULE: INITIATION FACTOR 2B;
15:	1iqp-A	3.5	3.7	70	326	7	PDB	MOLECULE: RFCS;
16|	4ph0-C	3.5	4.6	101	199	8	PDB	MOLECULE: BLV CAPSID;
17|	4ph0-D	3.5	4.2	101	198	8	PDB	MOLECULE: BLV CAPSID;
18|	4ph2-B	3.5	3.3	69	127	7	PDB	MOLECULE: BLV CAPSID - N-TERMINAL DOMAIN;
19:	1sxj-B	3.4	3.5	65	316	3	PDB	MOLECULE: ACTIVATOR 1 95 KDA SUBUNIT;
20:	2afd-A	3.4	2.7	59	88	14	PDB	MOLECULE: PROTEIN ASL1650;
21:	2yhe-A	3.4	2.8	40	639	8	PDB	MOLECULE: SEC-ALKYL SULFATASE;
22:	4u8y-B	3.4	4.4	64	1033	3	PDB	MOLECULE: MULTIDRUG EFFLUX PUMP SUBUNIT ACRB;
23:	2yhe-C	3.4	7.1	70	634	6	PDB	MOLECULE: SEC-ALKYL SULFATASE;
24:	2yhe-E	3.4	6.6	72	634	7	PDB	MOLECULE: SEC-ALKYL SULFATASE;
25|	4coc-B	3.4	3.1	64	79	9	PDB	MOLECULE: CAPSID PROTEIN P24;
26:	4av7-D	3.4	8.1	70	634	7	PDB	MOLECULE: SEC-ALKYLSULFATASE;
27:	4mbq-F	3.4	3.2	42	50	5	PDB	MOLECULE: MOTILITY PROTEIN FIMV;
28|	3g1g-B	3.4	2.9	61	75	8	PDB	MOLECULE: GAG POLYPROTEIN;
29:	2w8a-A	3.3	2.0	43	531	7	PDB	MOLECULE: GLYCINE BETAINE TRANSPORTER BETP;
30|	3ce7-A	3.3	2.7	59	90	5	PDB	MOLECULE: SPECIFIC MITOCHODRIAL ACYL CARRIER
31:	1eia-A	3.3	2.8	62	207	11	PDB	MOLECULE: EIAV CAPSID PROTEIN P26;
32:	1k6y-B	3.3	2.4	40	194	3	PDB	MOLECULE: INTEGRASE;
33:	2lo0-A	3.3	2.5	33	45	6	PDB	MOLECULE: UNCHARACTERIZED PROTEIN;
34:	3w9j-B	3.3	4.3	64	1030	5	PDB	MOLECULE: MULTIDRUG RESISTANCE PROTEIN MEXB;
35:	4dx6-B	3.3	4.6	67	1033	4	PDB	MOLECULE: ACRIFLAVINE RESISTANCE PROTEIN B;
36:	1iqp-D	3.3	3.9	70	326	7	PDB	MOLECULE: RFCS;

37:	2afe-A	3.3	2.8	59	88	12	PDB	MOLECULE: PROTEIN ASL1650;
38:	4av7-B	3.3	7.6	72	637	8	PDB	MOLECULE: SEC-ALKYLSULFATASE;
39:	1iqp-B	3.3	4.1	70	326	7	PDB	MOLECULE: RFCS;
40:	1k6y-D	3.3	3.7	52	189	2	PDB	MOLECULE: INTEGRASE;
41:	1k6y-C	3.3	3.3	49	191	4	PDB	MOLECULE: INTEGRASE;
42:	1k6y-A	3.3	4.2	54	192	2	PDB	MOLECULE: INTEGRASE;
43:	2lo0-B	3.3	2.9	36	45	6	PDB	MOLECULE: UNCHARACTERIZED PROTEIN;
44|	1afv-B	3.3	3.6	86	151	15	PDB	MOLECULE: HUMAN IMMUNODEFICIENCY VIRUS TYPE 1
45:	2o98-A	3.3	12.4	74	234	11	PDB	MOLECULE: 14-3-3-LIKE PROTEIN C;
46|	4ph0-A	3.2	4.1	100	201	9	PDB	MOLECULE: BLV CAPSID;
47|	1l6n-A	3.2	9.7	88	288	13	PDB	MOLECULE: GAG POLYPROTEIN;
48:	3d5l-A	3.2	9.1	68	203	7	PDB	MOLECULE: REGULATORY PROTEIN RECX;
49:	1mw7-A	3.2	2.3	41	220	10	PDB	MOLECULE: HYPOTHETICAL PROTEIN HP0162;
50:	2lni-A	3.2	3.5	43	133	2	PDB	MOLECULE: STRESS-INDUCED-PHOSPHOPROTEIN 1;
51:	2kc7-A	3.2	3.1	40	99	10	PDB	MOLECULE: BFR218_PROTEIN;
52:	4dx5-B	3.2	4.6	65	1033	3	PDB	MOLECULE: ACRIFLAVINE RESISTANCE PROTEIN B;
53|	4coc-C	3.2	2.7	59	73	5	PDB	MOLECULE: CAPSID PROTEIN P24;
54|	4coc-A	3.2	2.7	61	75	5	PDB	MOLECULE: CAPSID PROTEIN P24;
55|	4cop-B	3.2	2.7	57	68	5	PDB	MOLECULE: CAPSID PROTEIN P24;
56|	3lry-A	3.2	2.9	62	71	8	PDB	MOLECULE: HIV-1 CAPSID PROTEIN;
57|	3lry-B	3.2	2.8	61	71	8	PDB	MOLECULE: HIV-1 CAPSID PROTEIN;
58|	4ph0-B	3.2	4.2	88	188	9	PDB	MOLECULE: BLV CAPSID;
59|	1afv-A	3.2	3.6	86	151	15	PDB	MOLECULE: HUMAN IMMUNODEFICIENCY VIRUS TYPE 1
60|	2gon-C	3.2	4.3	85	138	14	PDB	MOLECULE: CAPSID PROTEIN P24 (CA);
61|	4ph3-B	3.2	3.1	64	115	8	PDB	MOLECULE: BLV CAPSID;
62:	3ual-A	3.2	12.1	69	230	7	PDB	MOLECULE: 14-3-3 PROTEIN EPSILON;
63:	1o9e-A	3.2	12.2	70	231	11	PDB	MOLECULE: 14-3-3-LIKE PROTEIN C;
64:	1o9c-A	3.2	12.3	69	231	10	PDB	MOLECULE: 14-3-3-LIKE PROTEIN C;
65:	3cu8-A	3.2	11.3	68	229	9	PDB	MOLECULE: 14-3-3 PROTEIN ZETA/DELTA;
66:	3ubw-A	3.2	12.1	69	230	7	PDB	MOLECULE: 14-3-3 PROTEIN EPSILON;
67:	1o9f-A	3.2	12.2	71	231	8	PDB	MOLECULE: 14-3-3-LIKE PROTEIN C;
68:	4fj3-A	3.2	12.2	70	220	9	PDB	MOLECULE: 14-3-3 PROTEIN ZETA/DELTA;
69|	3dph-B	3.2	2.9	63	80	10	PDB	MOLECULE: HIV-1 CAPSID PROTEIN;
70|	3ds3-B	3.2	2.6	59	73	10	PDB	MOLECULE: HIV-1 CAPSID PROTEIN;
71|	4ph1-A	3.2	3.2	63	73	6	PDB	MOLECULE: BLV CAPSID;
72|	2y4z-A	3.1	3.8	68	135	10	PDB	MOLECULE: CAPSID PROTEIN P30;
73|	5a9e-A	3.1	3.1	61	254	3	PDB	MOLECULE: DELTAMBD GAG PROTEIN;
74:	2liu-A	3.1	2.9	58	99	7	PDB	MOLECULE: CURA;
75:	2o98-B	3.1	2.8	42	237	12	PDB	MOLECULE: 14-3-3-LIKE PROTEIN C;
76:	3ph0-C	3.1	3.3	42	53	14	PDB	MOLECULE: ASCE;
77:	1s7e-A	3.1	2.0	40	147	10	PDB	MOLECULE: HEPATOCYTE NUCLEAR FACTOR 6;
78:	4dx7-B	3.1	4.5	65	1033	5	PDB	MOLECULE: ACRIFLAVINE RESISTANCE PROTEIN B;
79|	3ds2-B	3.1	3.2	70	84	11	PDB	MOLECULE: HIV-1 CAPSID PROTEIN;
80|	2eia-B	3.1	2.9	61	204	11	PDB	MOLECULE: EIAV CAPSID PROTEIN P26;
81|	4m0i-A	3.1	2.9	61	71	8	PDB	MOLECULE: HIV-1 CAPSID PROTEIN;
82|	4u0d-L	3.1	3.9	83	191	14	PDB	MOLECULE: GAG POLYPROTEIN;

```
 83|  4ph3-A  3.1  3.1   65   115    8 PDB  MOLECULE: BLV CAPSID;
 84:  4wrq-A  3.1 12.3   71   220    8 PDB  MOLECULE: 14-3-3 PROTEIN ZETA/DELTA;
 85:  1o9d-A  3.1 11.4   68   230    9 PDB  MOLECULE: 14-3-3-LIKE PROTEIN C;
 86:  4n84-B  3.1 12.5   68   226    9 PDB  MOLECULE: 14-3-3 PROTEIN ZETA/DELTA;
 87:  4fl5-B  3.1 12.2   69   229    9 PDB  MOLECULE: 14-3-3 PROTEIN SIGMA;
 88|  1a43-A  3.1  2.9   61    72    8 PDB  MOLECULE: HIV-1 CAPSID;
 89:  2c63-D  3.1 10.9   63   233   10 PDB  MOLECULE: 14-3-3 PROTEIN ETA;
 90:  4n7g-A  3.1 12.4   69   229    7 PDB  MOLECULE: 14-3-3 PROTEIN ZETA/DELTA;
 91|  1baj-A  3.1  2.8   60    71    8 PDB  MOLECULE: GAG POLYPROTEIN;
 92:  3ph0-D  3.1  3.8   48    53   15 PDB  MOLECULE: ASCE;
 93:  3uzd-A  3.1 12.2   69   229   13 PDB  MOLECULE: 14-3-3 PROTEIN GAMMA;
 94|  4ph1-C  3.1  3.1   64    79    6 PDB  MOLECULE: BLV CAPSID;
 95:  4n84-A  3.1 12.4   71   227    8 PDB  MOLECULE: 14-3-3 PROTEIN ZETA/DELTA;
 96:  3rmr-A  3.0  4.9   99   236   11 PDB  MOLECULE: AVIRULENCE PROTEIN;
 97:  2l0q-A  3.0  3.1   53    80    6 PDB  MOLECULE: ACYL CARRIER PROTEIN;
 98:  2br9-A  3.0  2.8   42   230   12 PDB  MOLECULE: 14-3-3 PROTEIN EPSILON;
 99:  2btp-A  3.0  2.8   42   248   14 PDB  MOLECULE: 14-3-3 PROTEIN TAU;
100:  3axy-C  3.0  2.8   42   235   12 PDB  MOLECULE: PROTEIN HEADING DATE 3A;
   :
101|  1qrj-A  3.0  3.1   64   214    9 PDB  MOLECULE: HTLV-I CAPSID PROTEIN;
   :
104|  4xfx-A  3.0  4.0   82   216   15 PDB  MOLECULE: HIV-1 CAPSID PROTEIN;
105|  4u0d-F  3.0  3.9   83   211   14 PDB  MOLECULE: GAG POLYPROTEIN;
106|  2jpr-A  3.0  4.4   83   145   14 PDB  MOLECULE: GAG-POL POLYPROTEIN;
107|  4xfz-A  3.0  3.1   79   213   15 PDB  MOLECULE: HIV-1 CAPSID PROTEIN;
108|  2l6e-A  3.0  3.9   62    94    8 PDB  MOLECULE: CAPSID PROTEIN P24;
109|  4xfy-A  3.0  3.9   83   216   14 PDB  MOLECULE: HIV-1 CAPSID PROTEIN;
110|  4u0d-G  3.0  3.9   82   212   15 PDB  MOLECULE: GAG POLYPROTEIN;
111|  4u0d-B  3.0  4.0   83   207   14 PDB  MOLECULE: GAG POLYPROTEIN;
   :
141|  4cop-A  2.9  2.9   64    83    8 PDB  MOLECULE: CAPSID PROTEIN P24;
142|  2xv6-C  2.9  2.8   55    68    5 PDB  MOLECULE: CAPSID PROTEIN P24;
143|  4u0c-A  2.9  4.0   83   210   14 PDB  MOLECULE: CAPSID PROTEIN P24;
144|  4u0d-H  2.9  4.2   83   204   14 PDB  MOLECULE: GAG POLYPROTEIN;
145|  2pwo-B  2.9  5.4   83   143   14 PDB  MOLECULE: GAG-POL POLYPROTEIN (PR160GAG-POL);
146|  2pwm-E  2.9  5.7   83   145   14 PDB  MOLECULE: GAG-POL POLYPROTEIN;
147:  3upv-A  2.9  7.1   59   125    7 PDB  MOLECULE: HEAT SHOCK PROTEIN STI1;
148|  4u0d-I  2.9  3.9   83   196   14 PDB  MOLECULE: GAG POLYPROTEIN;
149|  4u0d-A  2.9  4.2   83   216   14 PDB  MOLECULE: GAG POLYPROTEIN;
150|  3p05-E  2.9  3.2   78   199   14 PDB  MOLECULE: HIV-1 CA;
   :
165|  3ds4-B  2.9  3.0   63    75   10 PDB  MOLECULE: HIV-1 CAPSID PROTEIN;
166|  3ds4-A  2.9  2.8   59    77   10 PDB  MOLECULE: HIV-1 CAPSID PROTEIN;
   :
179|  3ds2-A  2.9  3.4   68    84   12 PDB  MOLECULE: HIV-1 CAPSID PROTEIN;
   :
```

```
183|  3ds3-A  2.9  2.7   58    73   10 PDB  MOLECULE: HIV-1 CAPSID PROTEIN;
   :
186|  2x82-A  2.8  3.3   67   145    9 PDB  MOLECULE: CAPSID PROTEIN P24;
   :
200|  3ds0-A  2.8  3.5   63    82   10 PDB  MOLECULE: HIV-1 CAPSID PROTEIN;
201|  2xv6-A  2.8  2.9   60    75   10 PDB  MOLECULE: CAPSID PROTEIN P24;
   :
204|  3mge-A  2.8  4.0   81   204   15 PDB  MOLECULE: CAPSID PROTEIN P24;
205|  1ak4-C  2.8  4.7   82   145   15 PDB  MOLECULE: CYCLOPHILIN A;
206|  2pwm-H  2.8  5.4   81   145   15 PDB  MOLECULE: GAG-POL POLYPROTEIN;
207|  4u0d-J  2.8  4.2   83   204   14 PDB  MOLECULE: GAG POLYPROTEIN;
   :
208|  1m9x-G  2.8  4.7   83   146   14 PDB  MOLECULE: CYCLOPHILIN A;
209|  1e6j-P  2.8  3.1   78   209   15 PDB  MOLECULE: IMMUNOGLOBULIN;
210|  4ph0-E  2.8  4.3   98   193    8 PDB  MOLECULE: BLV CAPSID;
   :
211|  4hkc-A  2.8 12.1   69   229    9 PDB  MOLECULE: 14-3-3 PROTEIN ZETA/DELTA;
212|  3axy-J  2.8 12.1   71   234    8 PDB  MOLECULE: PROTEIN HEADING DATE 3A;
213|  2c74-B  2.8 12.3   70   234   10 PDB  MOLECULE: 14-3-3 PROTEIN ETA;
214|  4o46-C  2.8 12.2   69   235   12 PDB  MOLECULE: 14-3-3 PROTEIN GAMMA;
215|  3axy-D  2.8 12.1   71   235    8 PDB  MOLECULE: PROTEIN HEADING DATE 3A;
216|  3ds1-A  2.8  2.7   57    81    5 PDB  MOLECULE: HIV-1 CAPSID PROTEIN;
   :
241|  2pwm-D  2.7  5.4   82   145   13 PDB  MOLECULE: GAG-POL POLYPROTEIN;
242|  4u0d-K  2.7  4.0   83   204   14 PDB  MOLECULE: GAG POLYPROTEIN;
   :
244|  2kod-A  2.7  3.6   65    88    5 PDB  MOLECULE: HIV-1 CA C-TERMINAL DOMAIN;
   :
249|  2v4x-A  2.6  3.4   68   131    9 PDB  MOLECULE: CAPSID PROTEIN P27;
   :
272|  3j34-H  2.6  3.6   82   231   15 PDB  MOLECULE: CAPSID PROTEIN;
   :
274|  2jyl-A  2.6  6.8   64    84    9 PDB  MOLECULE: CAPSID PROTEIN P24 (CA);
   :
363|  3j34-d  2.4  3.9   83   231   13 PDB  MOLECULE: CAPSID PROTEIN;
   :
409|  3j34-6  2.3  4.4   84   231   14 PDB  MOLECULE: CAPSID PROTEIN;
410|  3j34-Z  2.3  4.3   84   231   13 PDB  MOLECULE: CAPSID PROTEIN;
   :
471|  4e91-A  2.2  3.0   75   131   15 PDB  MOLECULE: GAG PROTEIN;
472|  3j34-c  2.2  4.4   82   231   16 PDB  MOLECULE: CAPSID PROTEIN;
473|  3j34-E  2.2  4.9   84   231   15 PDB  MOLECULE: CAPSID PROTEIN;
474|  3j34-A  2.2  3.6   83   231   16 PDB  MOLECULE: CAPSID PROTEIN;
   :
548|  1u7k-A  2.1  4.9   79   131    8 PDB  MOLECULE: GAG POLYPROTEIN;
549|  4ph0-F  2.1  4.5   97   199    8 PDB  MOLECULE: BLV CAPSID;
550|  3j34-i  2.1  4.9   81   231   16 PDB  MOLECULE: CAPSID PROTEIN;
```
